# Anatomy of the Nervous System in *Chelifer cancroides* (Arachnida: Pseudoscorpiones) with a Distinct Sensory Pathway Associated with the Pedipalps

**DOI:** 10.3390/insects13010025

**Published:** 2021-12-24

**Authors:** Torben Stemme, Sarah E. Pfeffer

**Affiliations:** Institute of Neurobiology, Ulm University, Albert-Einstein-Allee 11, 89081 Ulm, Germany; sarah.pfeffer@uni-ulm.de

**Keywords:** chemosensation, mechanosensation, morphology, Chelicerata, olfaction, somatotopy, chemotopy, immunofluorescence, brain, afferents

## Abstract

**Simple Summary:**

Most arthropods (uniting animals such as the chelicerates, e.g., spiders and their kin, as well as millipedes, centipedes, crustaceans, and insects) have distinct sensory appendages at the second head segment, the so-called antennae. The Arachnida (e.g., spiders and scorpions) do not possess antennae, but have evolved highly specialized sensory organs on different body regions. However, very limited information is available concerning pseudoscorpions (false scorpions). These animals do not seem to possess such specialized structures, but show dominant, multifunctional appendages prior to the first walking leg, called pedipalps. Here, we investigate the neuronal pathway of these structures as well as general aspects of the nervous system. We describe new details of typical arthropod brain compartments, such as the arcuate body and a comparatively small mushroom body. Neurons associated with the pedipalps terminate in two regions in the central nervous system of characteristic arrangement: a glomerular and a layered center, which we interpret as a chemo- and a mechanosensory center, respectively. The centers, which fulfill the same function in other animals, show a similar arrangement. These similarities in the sensory systems of different evolutionary origin have to be interpreted as functional prerequisites. Identifying these similarities helps to understand the general functionality of sensory systems, not only within arthropods.

**Abstract:**

Many arachnid taxa have evolved unique, highly specialized sensory structures such as antenniform legs in Amblypygi (whip spiders), for instance, or mesosomal pectines in scorpions. Knowledge of the neuroanatomy as well as functional aspects of these sensory organs is rather scarce, especially in comparison to other arthropod clades. In pseudoscorpions, no special sensory structures have been discovered so far. Nevertheless, these animals possess dominant, multifunctional pedipalps, which are good candidates for being the primary sensory appendages. However, only little is known about the anatomy of the nervous system and the projection pattern of pedipalpal afferents in this taxon. By using immunofluorescent labeling of neuronal structures as well as lipophilic dye labeling of pedipalpal pathways, we identified the arcuate body, as well as a comparatively small mushroom body, the latter showing some similarities to that of Solifugae (sun spiders and camel spiders). Furthermore, afferents from the pedipalps terminate in a glomerular and a layered neuropil. Due to the innervation pattern and structural appearance, we conclude that these neuropils are the first integration centers of the chemosensory and mechanosensory afferents. Within Arthropoda, but also other invertebrates or even vertebrates, sensory structures show rather similar neuronal arrangement. Thus, these similarities in the sensory systems of different evolutionary origin have to be interpreted as functional prerequisites of the respective modality.

## 1. Introduction

The arachnid order of pseudoscorpions comprises more than 3300 valid species [1]. Although their general morphology appears rather conserved, pseudoscorpions display an impressive complexity in terms of behavioral patterns and life styles. For example, they can live solitary, in loose groups, or even in social communities sharing food and hunting cooperatively (e.g. [2,3,4,5,6]). Furthermore, pseudoscorpions show different mating strategies, from deposition and collection of spermatophores without the presence of mates to complex courtship dances (e.g. [3,7]). A very fascinating behavior performed by many species is called phoresy, were individuals hitchhike on larger species by grasping the carrier with their pedipalps (e.g. [8,9]).

These and other complex behavioral capacities may depend on a well-elaborated chemosensory perception. However, chemosensation in pseudoscorpions has not been addressed to a sufficient degree. Evidence from behavioral observations indicates involvement of chemosensory cues in various aspects of pseudoscorpion ecology. It has been suggested that olfactory cues play an important role in phoretic behavior [10,11]. Furthermore, chemosensory cues seem to be essential in mating. It has been hypothesized that both males and females deposit and detect chemical cues on their mates [12,13]. In those species, where males do not guide females to spermatophore deposits, males attract females probably using pheromones [7,14]. These chemical cues are presumably released by special organs, called rams horn organs [3,7,15], a structure that is also evident in the here studied species, *Chelifer cancroides* [16]. Additionally, males seem to coat the spermatophores with a droplet containing pheromones, at least in those species where sperm packages are deposited in absence of females [17,18,19]. Along these lines, Judson suggested a pheromonal function of the sternal gland secretion, marking territories or spermatophores [20]. Vibration of the third walking legs during mating in males of *Withius piger* might distribute the chemical cue [7,20]. Associated with a complex courtship, males of social species—including *C. cancroides*—border a specific territory, which they defend from other males and in which they court visiting females [7]. These territories are hypothesized to be marked by chemosensory cues, as males persistently rub their ventral side against the substrate within these areas [7]. Finally, chemosensory cues might also play a role in finding prey, although no information has been published to our knowledge.

**Figure 1:**
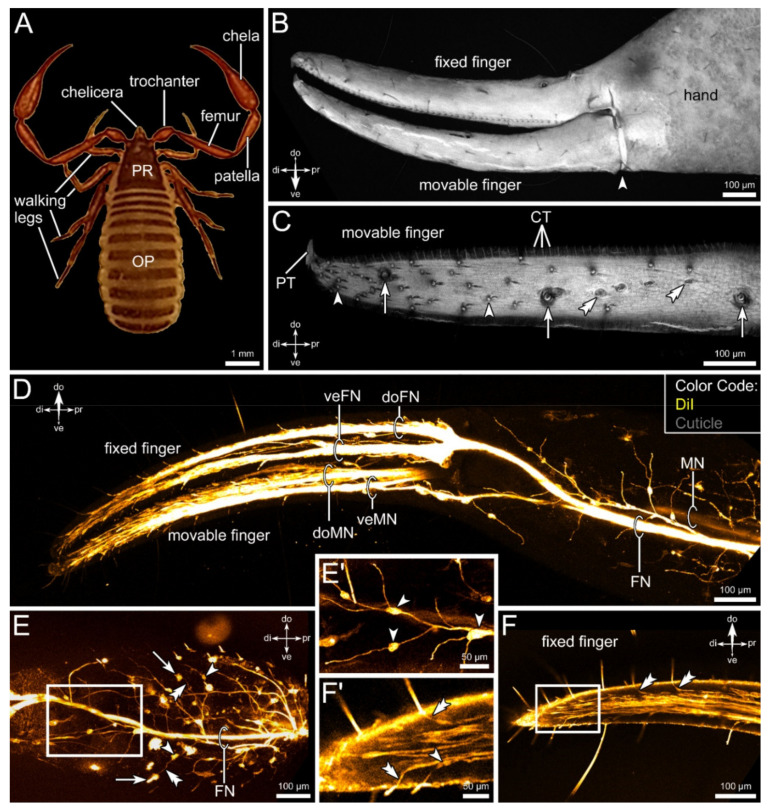
General morphology, the sensory equipment, and neuronal innervation of the pedipalps of *Chelifer cancroides*. (**A**) Dorsal view of the animal, showing the four pairs of walking legs, the chelicera, and the large pedipalps, consisting of the trochanter, femur, patella, and the chela (tibia + tarsus). All appendages are associated with the prosoma (PR). The opisthosoma (OP) does not possess any appendages. (**B**) Overview of the chelal surface stained with Congo red (gray). The chela possesses two fingers, a dorsal fixed finger, and a ventral movable finger (joint indicated by arrowhead). (**C**) Detail of the movable finger, showing the sensory equipment with different types of sensilla, e.g., trichobothria (arrows), shorter hair-sensilla (double arrowheads), and club-shaped sensilla (arrowheads). The chelal teeth (CT) and poison tooth (PT) are indicated. (**D**) Overview of the innervation of the chela based on lipophilic dye injection (DiI, yellow). Within the chelal hand, two main fibers can be distinguished, each innervating one of the fingers (FN: fixed finger nerve; MN: movable finger nerve). Shortly after entering the fingers, these nerves split into two branches (veFN, doFN: ventral and dorsal nerve of fixed finger; veMN, doMN: ventral and dorsal nerve of movable finger). (**E**,**E’**) Details of the innervation of the chelal hand. From the main nerve branches (FN shown), various branches split of and send fine branches (double arrowheads in (**E**)) toward sensory structures at the cuticle surface (arrows in (**E**)). Along these fine fibers, swellings of cell bodies can be observed (arrowheads in (**E**,**E’**)). (**F**,**F’**) Details of the innervation of the fixed finger. From the dorsal and ventral nerve branches within the fingers, numerous fibers branch off and innervate sensory structures (double arrowheads in (**F**,**F’**)). Again, swellings consisting of cell bodies are present (arrowhead in (**F’**)). Other abbreviations: di: distal; do: dorsal; pr: proximal; ve: ventral.

Although all these observations point to a well-developed chemosensory system, no clear evidence on a chemosensory organ nor special sensilla exists. Interestingly, some sensilla on the fingers and chelae are often termed ‘chemosensory setae’ in taxonomic contributions (e.g. [21,22,23,24,25,26]), but their ultrastructure has never been investigated. While other arachnid taxa have evolved special chemosensory detectors—such as antenniform legs in Amblypygi, Thelyphonida, or Opiliones [27,28,29,30], the so-called pectines in scorpions [31,32,33], or the fan-shaped malleoli in Solifugae (sun spiders and camel spiders) [34]—pseudoscorpions do not possess such conspicuous structures, at least at first glance. Promising candidates for such a primary chemosensory organ are the pedipalpal appendages, due to its elongated, anteriorly outreaching nature and their dense coverage by sensory sensilla of diverse types [3]. Beside mechanosensory trichobothria and proprioceptive lyriform organs, some of these sensilla are suggested to function as chemosensory detectors [3]. The pedipalps possess sophisticated chelae, which are built by the swollen tibia (also termed ‘hand’) extending into the fixed finger, and the tarsus forming the movable finger (see Figure 1A). The pedipalps do not only play a role in capturing and manipulating prey items (many pseudoscorpions possess venom glands at the tips of the fingers) but are also involved in social interactions [3].

Similar to the peripheral nervous system and its sensory equipment, the central nervous system of pseudoscorpions has received little attention as well. In early accounts, a rather simplified nervous system with a general anatomy of a fused ganglionic mass surrounding the esophagus, the so-called synganglion [35], has been described [3,36,37,38,39]. Besides the clearly distinguishable arcuate body (an unpaired midline neuropil presumably involved in coordination of limb motor control; e.g. [40,41]), only residues of mushroom bodies (higher-order integration centers in arthropod brains involved in multimodal integration, learning, and memory; e.g. [42,43,44,45,46]) have been identified in the protocerebrum [3,39]. Lehmann and Melzer [47] re-visited the morphology of a rather simple visual system of *Neobisium carcinoides*, updating pioneering studies by Hanström [39] and Boissin and Cazal [48]. Finally, immunocytochemical investigations and identification of neuroactive compounds are limited to a recent study, addressing the histaminergic system in *C. cancroides* [49].

Intriguingly, Hanström (1919) identified a neuropil consisting of spherical subunits, which he termed ‘glomerular mass’ (German: ‘*Glomerulimasse*’) [38]. These glomeruli extend on both hemispheres adjacent to the esophagus at the transition between the brain and subesophageal neuromeres [38,39,50]. However, the association of these glomeruli to a specific appendage or neuromere remains unresolved (compare [38,39,50]). Interestingly, glomerular structures within the central nervous system are a rather strong indicator for chemosensory processing, as these conspicuous structures are found in chemosensory neuropils in many invertebrate but also vertebrate representatives (reviewed in, e.g. [51,52,53]). It should be emphasized, that glomerular neuropils are also found in other neuronal regions than chemosensory pathways, namely, in protocerebral circuits associated with the visual or mushroom body pathways (see discussion) (e.g. [54,55,56,57,58,59,60]).

The present contribution sets out to revisit the general morphology of the central nervous system of the pseudoscorpion *C. cancroides* with immunocytochemical methods combined with lipophilic dye labeling and confocal laser-scanning microscopy. In order to answer the question, if the glomerular mass identified by previous contributions [38,39,50] is associated with the pedipalps, we focus on the neuronal pathway of these appendages. In the following, we present first morphological evidence of a chemosensory pathway associated with the pedipalps in the pseudoscorpion *C. cancroides*.

## 2. Materials and Methods

### 2.1. Animal Collection

Adults of *Chelifer cancroides* (both sexes) were collected from an old hayloft near Rinteln (Lower Saxony, Germany). After collection, animals were kept in jars with crumpled paper as hiding place for transport to the laboratory where they were sacrificed as soon as possible.

Before dissection, animals were anesthetized with 99.7 vol% CO_2_ and cooled down in the refrigerator at approximately 4 °C for at least 10 min. Afterwards, specimens were viewed with a dissection microscope (Stemi 508, Zeiss, Oberkochen, Germany) ventral side up, in order to determine the sex: ventrally, males show dark bilateral symmetric structures—known as rams horn organs, which are lacking in females (description of sex-specific differences see [16]). Afterwards, animals were processed according to the different experiments.

### 2.2. Staining of Chelal Cuticle Using Congo Red

In order to visualize the sensory equipment of the chelal fingers, the chelae (12 female and 10 male chelae) were cleaned and treated with Congo red (Sigma-Aldrich, St. Louis, MO, USA). After anesthetizing the animals with CO_2_, the pedipalps were cut proximally to the patella-chela-joint and transferred to 70% ethanol (Merck, Darmstadt, Germany) for at least 24 h. Afterwards, the specimens were incubated in 5% potassium hydroxide (KOH; Merck) at room temperature for several days (protocol from [61]). Then, specimens were transferred to glacial acetic acid (Merck) for 15 min, followed by six washing steps of 15 min each in distilled water. In the next step, the chelae were incubated in Congo red dissolved in distilled water (1.5 mg Congo red per milliliter distilled water, see [62]) for two weeks. Finally, the preparations were washed several times in distilled water until no solute Congo red was visible anymore and mounted in 90% glycerin (Merck) in distilled water. To avoid squeezing of preparations hollow-ground microscopic slides (Paul Marienfeld GmbH, Lauda-Königshofen, Germany) were used.

### 2.3. Vibratome Sections, Immunohistochemistry, and Phalloidin Labeling

To describe the general anatomy of the nervous system, the synganglia of 11 females and 9 males were dissected and freed from surrounding tissue with fine forceps (all dissection tools obtained from Fine Science Tools, Heidelberg, Germany). Afterwards, the tissue was fixed in 4% paraformaldehyde (PFA; Sigma-Aldrich) dissolved in phosphate-buffered saline (PBS; 10 mM sodium phosphate, 150 mM sodium chloride, pH 7.4; chemicals obtained from Merck) over night at 4 °C. After three washing steps with PBS for at least 15 min each, the synganglia were transferred to black scale pans, carefully dried with filter paper, and briefly covered with poly-D-lysine (1 mg/mL in demin. H_2_O; Merck) to achieve better connection between the tissue and embedding medium. The removal of the poly-D-lysine was followed by embedding the tissue in 7% low-melting-point agarose (Carl Roth, Karlsruhe, Germany) dissolved in distilled water at approximately 35 °C. The preparations were cooled to 4 °C and the trimmed blocks were cut into 50 μm-thick sections in the horizontal, frontal, or sagittal plane by using a VT 1000 S Vibratome (Leica, Wetzlar, Germany).

The slices were permeabilized for 1 h in 0.3% saponin (Sigma Aldrich) in PBS containing 0.3% Triton X-100 (Merck) (PBS-TX 0.3%), washed three times for at least 15 min each in PBS-TX 0.3%, and incubated for at least 3 h in 5% normal goat serum (Vector Laboratories, Burlingame, CA, USA) in PBS-TX 0.3% as the blocking solution. In the following step, the primary antiserum mouse-anti-synapsin (Developmental Studies Hybridoma Bank, University of Iowa, IA, USA, 3C11; 1:40) were applied overnight at room temperature in blocking solution containing 1% Triton X-100. After three washing steps for 15 min in PBS-TX 0.3%, the preparations were incubated for at least 3 h at room temperature in Alexa Fluor 488-conjugated goat anti-mouse secondary antibody (Invitrogen, Carlsbad, CA, USA, Lot 1,907,294), diluted 1:250 in a blocking solution containing 0.3% Triton X-100. To visualize the filamentous actine, the immunocytochemical labeling was accompanied by treatment with AlexaFluor488-conjugated Phalloidin (Molecular Probes, Eugene, OR, USA) simultaneously. The methanolic stock solution was allowed to evaporate and then redissolved at 1:50 (equivalent to 4 U/mL) in a secondary antibody solution. Preparations were washed two times in PBS-TX 0.3% and once with PBS for 15 min each and finally mounted on adhesive microscope slides in Mowiol (Merck).

In order to visualize the position and relative size of the synganglion within the body, ten animals (3 females, 7 males) were processed slightly different to the previous protocol: five animals were cold anesthetized and legs as well as pedipalps were cut distally to the coxa. Furthermore, the posterior part has been cut posteriorly to the fourth walking leg. Afterwards, the specimens were incubated in 4% PFA for 48 h at 4 °C. After three washing steps in PBS, the specimens were embedded in 7% low-melting-point agarose as described above, and further sliced sagittally in 150 µm sections. The next steps correspond to the procedure describes above. In the remaining 5 specimens, the carapace was coarsely removed after fixation and processed as above, but without sectioning the specimens.

### 2.4. Lipophilic Dye Labeling of Pedipalpal Nerves

In order to visualize the innervation of the pedipalpal chela and the projection pattern of the associated afferents toward the central nervous system, the lipophilic tracers DiI (1,1′-Dioctadecyl-3,3,3′,3′-tetramethylindocarbocyanine perchlorate, Sigma-Aldrich) and/or DiA (4-(4-(Dihexadecylamino)styryl)-N-methylpyridinium iodide, ATT Bioquest, Biomol, Hamburg, Germany) were used.

Living specimens were anesthetized and the legs, opisthosoma, carapace, and tips of the chelae were removed in 18 animals (9 males, 9 females). In another 8 animals (4 males, 4 females), only the tips of the fixed and movable finger were cut. The chelae and bodies were fixed for 48 h at 4 °C in 4% PFA, followed by three washing steps with PBS for 30 min each. The broken tips of pulled glass micropipettes (Harvard Apparatus LTD, Holliston, MA, USA, type GC100TF–10) were submerged in methanolic dye solution. The methanol was allowed to evaporate, resulting in a thin coat of dye crystals on the micropipette [63]. The tip of these coated micropipettes was injected into (a) the isolated chelae; (b) the stumps of the pedipalps at the level of the patella; and (c) the movable and fixed fingers. The preparations were stored in PBS containing 0.1% sodium azide (Carl Roth) at approximately 25 °C in a dark place. The progress of dye travelling was checked using an upright fluorescent microscope (SliceScope Pro, Scientifica, Uckfield, UK) every two days.

Afterwards, the remains of the pedipalps were cut at the coxae and stored together with the isolated chelae for further processing (see below). The central nervous system was freed from the remaining surrounding tissue and further processed for fluorescent labeling of the synapsin and actin, and for the nuclear counterstain. The protocol for sectioning and immunolabeling followed the descriptions above, with the following differences: permeabilization with saponin and Triton-X100 was omitted to maintain the stability of membranes and to avoid fading of the lipophilic tracer labeling.

Pedipalps and chelae treated with lipophilic dyes were incubated in 70% ethanol for a maximum of 30 min in order to remove dye crystals, which might have attached to the cuticle. Afterwards, the pedipalps and chelae were incubated in 50% and subsequently 90% glycerol in PBS for approx. 3 h and mounted in fresh 90% glycerol in PBS.

### 2.5. Antibody Characterization

A monoclonal mouse anti-*Drosophila* synapsin antibody (“SYNORF1”, Developmental Studies Hybridoma Bank) raised against a *Drosophila* GST-synapsin fusion protein was applied. This antibody reacts with a highly conserved epitope, as it labels neuropil structures over a wide range of arthropod taxa (e.g. [64,65,66,67]), including arachnid representatives, e.g., spiders [58,68,69], scorpions [49,70], and amblypygids (whip spiders) [58]. This antibody has also been used as a structural marker in the focus species of this contribution, *C. cancroides* [49]. In Western blots of brain tissues of *Drosophila* and the crustacean *Coenobita clypeatus* identical bands were stained by the synapsin antibody, which suggests that the epitope for SYNORF1 is strongly conserved between *Drosophila* and *Coenobita* [64].

### 2.6. Microscopy and Image Acquisition

Sections were examined with a Leica TSC SP5 II confocal microscope (cLSM). Z-series were processed with NIH ImageJ, v. 1.8 (Rasband WS, ImageJ, U.S. National Institutes of Health, Bethesda, MD, USA, http://rsb.info.nih.gov/ij/ (accessed on 20 November 2021)), producing maximum projections. Image processing and panel preparation were conducted with Adobe Photoshop 6.0 (San Jose, CA, USA), including global contrast and brightness adjustment.

## 3. Results

### 3.1. External Morphology and Innervation Pattern of the Pedipalps

From proximal to distal, the pedipalps of pseudoscorpions consist of the coxa, trochanter, femur, patella, and the chela (terminology from [16]) (Figure 1A). The latter is built up of the tibial hand, which terminates in a fixed (belonging to the tibia) and a movable finger (tarsus) (Figure 1B). The movable finger is positioned ventrally, the fixed finger dorsally. The fingers are slightly bent toward the body (Figure 1A,B).

Both fingers possess numerous sensory sensilla of various forms, including trichobothria and short hair-sensilla as well as small club-shaped structures (Figure 1C). The sensilla are distributed on the entire surface of both fingers but seem to increase in number from proximal to distal (Figure 1C). These sensilla are innervated by a large number of sensory neurons (Figure 1D,F,F’). Directly after entering the chela, the pedipalpal nerve splits into two main branches, each supplying a specific finger: the fixed finger nerve and the movable finger nerve (Figure 1D,E). Thinner branches split off and are associated with structures on the cuticle of the hand, such as, e.g., sensory sensilla or glands (Figure 1D,E,E’). Swellings on these fine tracts represent clusters of cell somata (arrowheads in Figure 1E,E’). Additionally, some thinner tracts enter the chela and form a fine net of neuronal processes, which are not associated with cuticular structures, but probably represent motoneurons. Within both fingers, the nerve tracts split up again at the base of the fingers (Figure 1D). One branch innervates the sensilla positioned on the dorsal portion of the fingers, while the other tract is associated with the sensilla on the ventral part (Figure 1D). Toward the tip of the fingers, numerous fine branches leave the main tracts, innervating the sensory structures (Figure 1F). Close to the sensilla, clusters of somata can be observed, from which the dendrites reach out toward the base of the sensilla (Figure 1F,F’).

### 3.2. General Anatomy of the Central Nervous System

**Figure 2:**
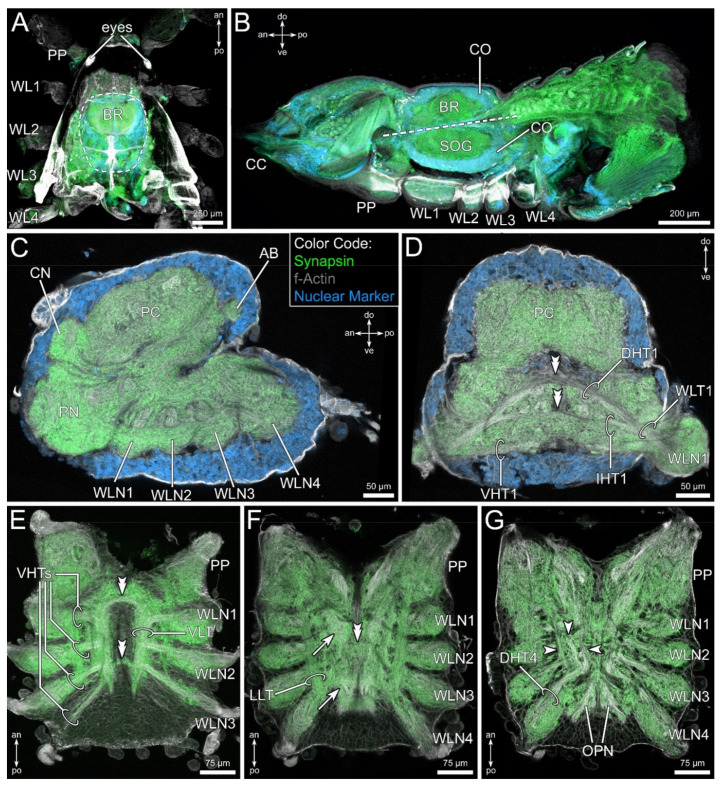
General morphology of the central nervous system of *Chelifer cancroides*, revealed by fluorescent labeling of synaptic regions (green), f-actin (gray), and cell bodies (blue). (**A**) Position of the synganglion within the prosoma from a dorsal view. The dashed line indicates the outline of the synganglion. (**B**) From a sagittal view at the midline level, the synganglion is positioned at the dorsal margin of the prosoma. The soma cortex (CO) surrounds the neuropilar regions. The synganglion is divided into a supraesophageal brain (BR) and the subesophageal ganglion (SOG). Dashed line indicates the esophagus. (**C**) Sagittal slice of the synganglion at a more lateral level compared to (**B**). Ventrally, the four walking leg neuromeres (WLN1–4) can be recognized. Anterior to the WLN lies the large pedipalpal neuromere (PN). Dorsally to the PN, the cheliceral neuromere (CN) can be distinguished. The main part of the brain consists of the protocerebrum (PC), including the dorso-posterior arcuate body (AB). (**D**) Dorsal section of the synganglion at the level of the first walking leg neuromere (WLN1). A thick fiber tract (WLT1) connects the lateral parts of the WLN1 with medial areas of the synganglion. The WLT1 splits into three branches, a ventral (VHT1), an intermediate (IHT1) and a dorsal horizontal tract (DHT1). At least the IHT1 and the DHT1 seem to contain contralateral projections (double arrowheads). (**E**) Horizontal section of the synganglion at a ventral level. The ventral horizontal tracts of the walking legs (VHTs) are connected to a ventral longitudinal tract near the midline (VLT). Arrowheads point on two commissures formed by the bilateral symmetric VLTs. (**F**) At a more dorsal level, a thick, more lateral longitudinal tract (LLT) appears. Between these bilateral symmetric tracts, paired (arrows) and unpaired midline neuropils (double arrowhead) can be distinguished. (**G**) At an even more dorsal level, the dorsal horizontal tracts of the walking legs split up in several dorsal longitudinal tracts (arrows). Note the fused mass of opisthosomal ganglia (OPN), between the fourth walking leg neuromeres (WLN4). Other abbreviations: an: anterior; CC: chelicera; do: dorsal; po: posterior; PP: pedipalp; ve: ventral; WL1–4: walking legs 1–4.

**Figure 3:**
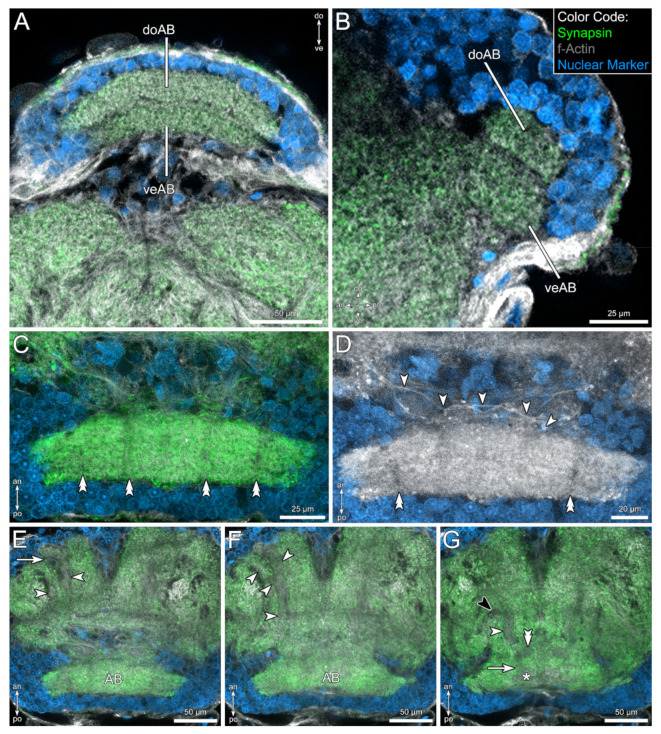
Neuroanatomical details of the protocerebrum in *Chelifer cancroides*, revealed by fluorescent labeling of synaptic regions (green), f-actin (gray), and cell bodies (blue). (**A**,**B**) Frontal (**A**) and sagittal view on the protocerebrum. The arcuate body (AB) is located at a postero-dorsal position and consists of a dorsal (doAB) and a ventral layer (veAB). (**C**) In horizontal view, the arcuate body is built up of several palisade-like subunits, which are separated by fiber tracts (double arrowheads in (**C**) and (**D**)). (**D**) Distinct fibers (arrowheads) connect the arcuate body and contralateral protocerebral neuropils. (**E**–**G**) Ventral to dorsal series of the protocerebrum, showing the components of the mushroom bodies. Two fiber tracts (arrowheads in (**E**)) emerge from an antero-lateral neuropil (arrow in (**E**)). More ventrally both fiber tracts (arrowheads in (**F**)) fuse and project further posteriorly. At a dorsal level, the fused main tract (white arrowhead in (**G**)) terminates in a postero-medial lobe (double arrowhead in (**G**)) close to the midline. A lateral tract leaves the main tract (black arrowhead in (**G**)). It seems, that the main tract projects further posteriorly (arrow in (**G**)) and terminates in a second lobe (asterisk in (**G**)) in close vicinity to the arcuate body. Other abbreviations: an: anterior; do: dorsal; po: posterior; ve: ventral.

The central nervous system of *C. cancroides* is positioned at the level of the first to third walking leg, directly beneath the dorsal cuticle (Figure 2A,B). As we could not identify any differences of the neuronal anatomy between males and females throughout our experiments, we do not distinguish between sexes in the following descriptions. The neuromeres are fused, forming a typical synganglion—connectives between the segmental neuromeres cannot be distinguished (Figure 2C–G). A contiguous soma cortex surrounds the fused central nervous system, and only few cell bodies are positioned within the neuropilar regions (Figure 2B–D). The synganglion can be divided into two main regions, the subesophageal ganglion and the supraesophageal ganglion, also called the brain (Figure 2B). The subesophageal ganglion is composed of the four walking leg neuromeres (Figure 2C). A small neuropilar region follows the fourth walking leg neuromeres posteriorly, which represents residuals of the condensed opisthosomal neuromeres (Figure 2G). Anteriorly, the first walking leg neuromeres are followed by the large pedipalpal neuromere (Figure 2C), which is built up of several distinct neuropils. However, their boarders to each other are difficult to outline. This neuropil spans laterally around the esophagus, and thus builds the connection between the subesophageal ganglion and the brain (Figure 2C). Dorsally to the pedipalpal neuropil, already belonging to the brain, the smaller cheliceral neuropil can be distinguished (Figure 2C). Finally, the prominent protocerebrum follows posterodorsally (Figure 2C,D and Figure 3).

**Figure 4:**
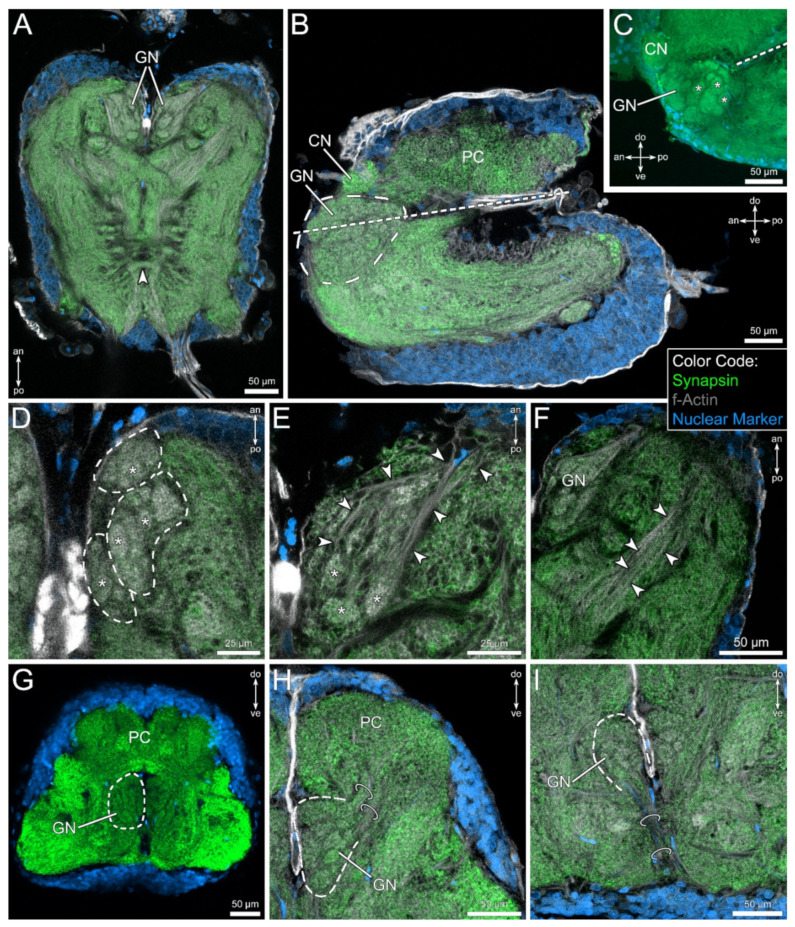
Position and morphology of glomerular structures in the pedipalpal neuropil, revealed by fluorescent labeling of synaptic regions (green), f-actin (gray), and cell bodies (blue). (**A**) In the most dorsal horizontal section (in relation of the subesophageal ganglion) of the synganglion, a conspicuous, drop-shaped neuropil (GN) consisting of glomerular subunits can be observed in antero-medial aspects of the pedipalpal neuromere. Note the commissure in posterior region (arrowhead). (**B**,**C**) In the sagittal view, the glomerular neuropil (GN) spans spherically around the esophagus, with a fraction lying dorsally, the other part lying ventrally with respect to the esophagus (dashed lines). Asterisks indicate glomeruli. (**D**) In the dorsal view, the arrangement of the glomeruli (asterisks) gives the impression of separated subcompartments (indicated by dashed lines). (**E**) The glomerular neuropil is penetrated by fibers (arrowheads) from the antero-lateral direction, innervating the glomeruli (asterisks). (**F**) Besides the glomerular neuropil, another distinct neuropil is found postero-laterally, having a stratified appearance of parallel fibers (arrowheads). (**G**–**I**) Frontal views on the glomerular neuropil (highlighted by dashed lines), showing a spheric to drop-shaped appearance. The glomerular neuropil is connected to dorsal, protocerebral regions by two distinct tracts (rings in (**H**)). Ventrally, fibers connect the glomerular neuropil to the ventral soma cortex (rings in (**I**)). Other abbreviations: an: anterior; CN: cheliceral neuropil; do: dorsal; PC: protocerebrum; po: posterior; ve: ventral.

Within the subesophageal ganglion, several horizontal and longitudinal tracts can be distinguished. The walking leg nerves enter the associated neuromere and split into three horizontal tracts: a ventral (VHT), an intermediate (IHT), and a dorsal horizontal tract (DHT) (Figure 2D,G). At least the prominent DHT and the smaller IHT closely approach the midline and seem to contain contralateral projections (double arrowheads in Figure 2D). The VHTs form a conspicuous, ventrally positioned longitudinal tract (VLT) near the midline (Figure 2E). The bilateral symmetric VLTs are connected anteriorly and posteriorly by two commissures (double arrowheads in Figure 2E). More dorso-laterally, another prominent longitudinal tract runs from the last walking leg neuropil to the pedipalpal neuromere (LLT, Figure 2F). In between the bilateral symmetric LLTs and dorsally to the VLTs, two types of neuropilar regions can be distinguished: paired medial neuropils (arrows in Figure 2F), and at least one unpaired median neuropil (double arrowhead in Figure 2F). Dorsally in the subesophageal ganglion, the DHTs split into at least three dorsal longitudinal tracts (arrows in Figure 2G). Furthermore, several commissural fibers connect the hemispheres of the subesophageal ganglion at a dorsal level (exemplified shown in Figure 4A).

The protocerebrum, although comparatively large, does not possess many distinct subcompartments. One exception is the rather isolated postero-dorsal arcuate body (Figure 2C and Figure 3). The arcuate body is slightly bent ventrally and consists of two distinct layers: a thicker ventral layer and a thinner dorsal layer (Figure 3A,B). The arcuate body consists of several palisade-like subunits, which are separated by horizontal fiber bundles (double arrowheads in Figure 3C,D). Furthermore, fibers associated with the arcuate body cross the midline anteriorly and connect the arcuate body to the adjacent lateral parts of the protocerebrum (arrowheads in Figure 3D).

Besides the arcuate body, a prominent tract can be distinguished in the protocerebrum, which we interpret as being part of the mushroom bodies. From a small, rather indistinct antero-lateral neuropil, two conspicuous tracts run posteriorly (Figure 3E). Shortly after leaving that neuropil, both tracts fuse and further project posteriorly (Figure 3F). This tract is associated with a medial mushroom body lobe close to the midline (Figure 3G). From here, the tract is difficult to follow, but it seems that it continues in a posterior direction and terminates in a second mushroom body lobe in direct vicinity of the ventral aspects of the arcuate body (Figure 3G). Finally, a lateral tract leaves the main tract (Figure 3G).

### 3.3. The Pedipalpal Neuropil Houses a Glomerular and a Stratified Neuropil

The pedipalpal neuropil is situated within the anterior part of the synganglion, directly lateral to the esophagus, connecting the subesophageal ganglion and the brain (Figure 2C). Apart from the protocerebrum, the pedipalpal neuropil is the largest neuromere within the nervous system of *C. cancroides*. The pedipalpal neuropil contains a conspicuous neuropilar region, which consists of numerous spheric or ovoid substructures of different size (asterisks in Figure 4C–E). This glomerular neuropil spans above and below the level of the esophagus (Figure 4B,C). The overall shape of this neuropil appears spheric to ovoid in the sagittal (Figure 4B,C) and frontal view (Figure 4G–I), but possesses at least three sublobes in the horizontal plane (Figure 4D). The glomerular neuropil is innervated by fibers coming from an anterolateral direction (arrowheads in Figure 4E). These fibers surround the glomerular neuropil and penetrate the glomeruli from the antero-lateral direction.

In a posterolateral position to the glomerular neuropil, another distinct region can be observed, receiving input from the same direction as the glomerular neuropil. It appears as an elongated neuropil, extending from an antero-lateral to a postero-medial position within the pedipalpal ganglion and possesses a stratified or lamellar organization (arrowheads in Figure 4F).

In the frontal view, two fiber bundles projecting in the dorso-lateral direction connect the glomerular neuropil with the protocerebral regions (Figure 4H). These tracts are assumed to consist of projection neurons. However, a clear destination within the protocerebrum could not be identified (see Section 4). Finally, distinct tracts connect the glomerular neuropil with the ventral soma cortex (Figure 4I), which we interpret as the projections of interneurons.

### 3.4. Lipophilic Dye Injection Reveal Connectivity of the Pedipalpal Nerve and Central Neuropilar Regions

**Figure 5:**
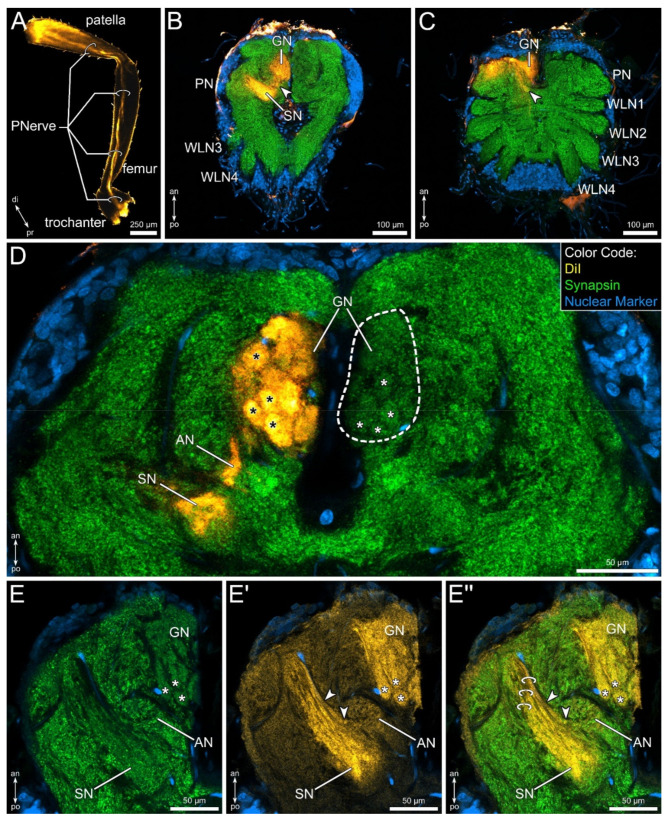
Innervation of the pedipalpal neuropil revealed by lipophilic dye labeling (DiI, yellow), injected at the distal part of the patella, combined with fluorescent labeling of the synaptic regions (green) and cell bodies (blue). (**A**) Overview of a dye injected pedipalp, visualizing the projection pattern of the pedipalpal nerve (PNerve). (**B**,**C**) Maximum projections of two consecutive horizontal sections, from ventral (**B**) to dorsal (**C**). Dye-labelled fibers enter the glomerular (GN) and the stratified neuropil (SN). Further, a small neuropil between GN and SN is labelled (arrowheads; accessory neuropil: AN). (**D**) Detail of the pedipalpal neuropil (PN), horizontal view. The glomeruli (asterisks) are clearly labelled. Furthermore, the accessory neuropil (AN) and the medial portion of the stratified neuropil (SN) are marked. Note that no labeling is evident in the contralateral GN (dashed line). (**E**,**E’**,**E”**) Horizontal section of the pedipalpal neuropil labelled against synapsin (**E**) and by lipophilic dye (**E’**); both channels merged in (**E”**). Glomeruli are indicated by asterisks. Note the layered innervation of the stratified neuropil, indicated by rings in (**E”**). The AN seems to receive input from anterior fibers associated with the SN (arrowheads in (**E’**,**E”**)). Other abbreviations: an: anterior; di: distal; po: posterior; pr: proximal; WLN1–4: walking leg neuromeres 1–4.

In order to verify if the glomerular and stratified neuropils are associated with the pedipalps, lipophilic dye injection in the distal part of the patella was performed. The course of the pedipalpal nerve toward the central nervous system can clearly be visualized (Figure 5A). Within the central nervous system, the tracer labels only structures within the pedipalpal neuromere (Figure 5B,C).

Three subregions of the pedipalpal neuropil are clearly labelled: the glomerular neuropil, the stratified neuropil, as well as a yet unidentified small neuropil, which we here term accessory neuropil (Figure 5B–E”). Within the glomerular neuropil, every glomerulus is labelled rather homogenously (Figure 5D). Additionally, the lamellae of the stratified neuropil show strong labeling (Figure 5E’,E”).

The accessory neuropil seems to receive input from fibers running parallel to the most anterior lamellae of the stratified neuropil (arrowheads in Figure 5E’,E”). Notably, no contralateral projections nor ipsilateral projections toward other neuromeres could be observed.

### 3.5. Differential Innervation by Afferents from the Fixed and Movable Fingers

**Figure 6:**
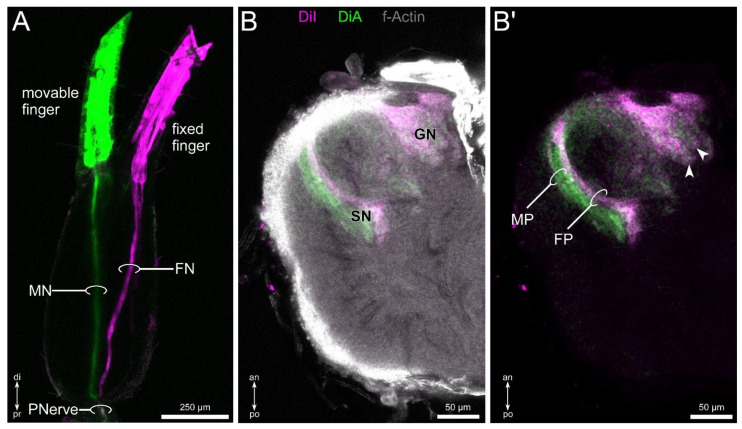
Differential labeling of movable and fixed finger nerves with lipophilic dyes (DiI: magenta; DiA: green), combined with f-actin labeling (gray). (**A**) Overview of dye-injected fingers, visualizing the projection pattern of the fixed finger (FN) and movable finger nerve (MN). At the chela–patella joint, both fibers fuse (pedipalpal nerve: PNerve). (**B**,**B’**) Horizontal view, showing the innervation pattern of the glomerular neuropil (GN) and the stratified neuropil (SN). While the GN is innervated homogenously (arrows point to selected glomeruli), the stratified neuropil shows a distinct innervation pattern by fibers associated with the movable finger (movable finger portion: MP) and fixed finger (fixed finger portion: FP). Other abbreviations: an: anterior; di: distal; po: posterior; pr: proximal.

**Figure 7:**
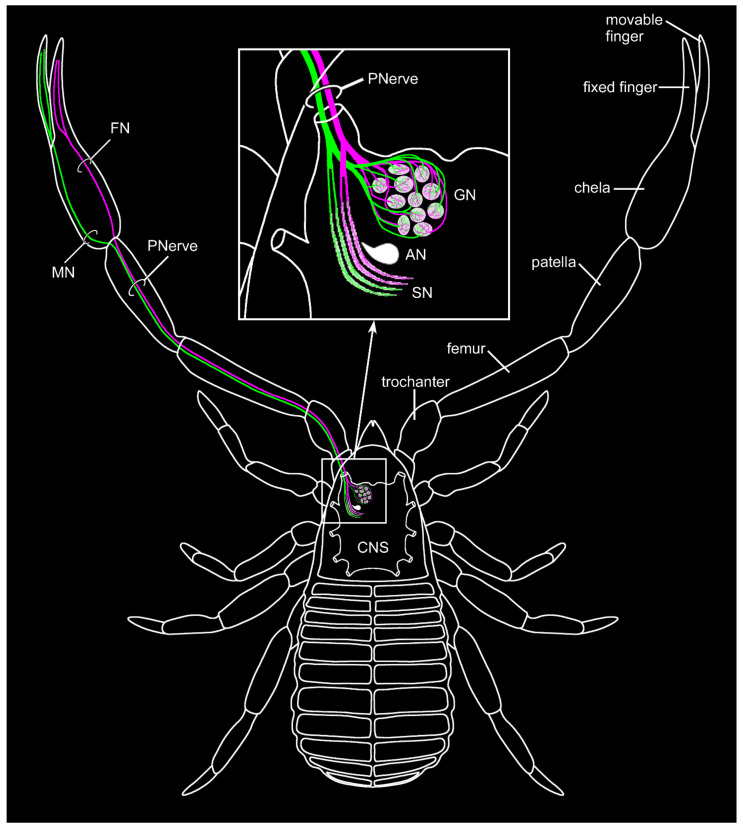
Schematic summary of the projection pattern of the sensory structures associated with the pedipalpal chelae of *Chelifer cancroides*. Differential labeling of afferents of the fixed (FN: fixed finger nerve, magenta) and movable finger (MN: movable finger nerve, green) innervate two neuropils in the pedipalpal neuromere within the central nervous system (CNS): The glomerular neuropil (GN) is innervated rather homogenously by projections from both fingers, while the stratified neuropil (SN) shows a clear separation in an anterior portion innervated by afferents from the fixed finger and a posterior portion innervated by fibers associated with the movable finger. Other abbreviations: AN: accessory neuropil; PNerve: pedipalpal nerve.

Differential lipophilic labeling of the fixed and movable finger revealed a distinct projection pattern. Within the hand, the two tracts originating in the fixed and movable finger project discretely toward the metatarsus–tibia joint (Figure 6A). Before entering the joint, the two nerves fuse and form the pedipalpal nerve, and follow the same path toward the pedipalpal neuropil. In the central nervous system, both tracers label the glomerular neuropil in a homogenous way (Figure 6B,B’). In contrast, the stratified neuropil shows a differential innervation by afferents associated with the fixed and movable finger. The sensilla associated with the latter innervates more the posterior regions, and sensilla on the fixed finger project to the anterior areas of the stratified neuropil (Figure 6B,B’). The characters of the pedipalpal sensory pathway are summarized in Figure 7.

## 4. Discussion

Most of our knowledge on the morphology of the nervous system in pseudoscorpions is based on older investigations with limited technical potential in comparison to current techniques [3,36,37,38,39]. Here, we presented a comprehensive description of the general organization of the central nervous system of *C. cancroides*, including details concerning the mushroom bodies and the arcuate body. Furthermore, we identified a glomerular and a stratified neuropil innervated by afferents from the pedipalps, revealing a sophisticated chemo- as well as mechanosensory pathway.

### 4.1. Comparative Aspects of Prominent Protocerebral Neuropils

#### 4.1.1. The Arcuate Body

In Arachnida, the arcuate body is a prominent midline neuropil in a postero-dorsal position (e.g. [35,39,54,71,72,73,74]), showing similar characteristics in all taxa: it appears as a crescent-shaped, layered neuropil with palisade-like arrangement. We could observe this general arrangement in the brain of *C. cancroides* as well (Figure 3). It has been suggested that this unpaired midline neuropil is involved in the coordination of limb motor control [40,41,75], although this hypothesis is mainly based on extrapolated results obtained from crustaceans and insects. Interestingly, the motoric repertoire seems to correlate with the elaboration and relative size of the arcuate body [40]. Besides several connections of the arcuate body to midbrain areas, it receives input from visual pathways, at least in spiders (e.g. [57,59,60,71]). In pseudoscorpions, Boissin and Cazal proposed a direct connection of the visual system to the arcuate body in *Hysterochelifer meridianus* [48]. However, Lehmann and Melzer found no evidence of a direct connection between the arcuate body and visual neuropils in another pseudoscorpion species, *N. carcinoides* [47]. We could not identify any tracts connecting the arcuate body with optic ganglia in *C. cancroides*. However, due to the simplicity of the visual pathway in combination with the applied methods, we were not able to address any details of the visual pathway in this contribution.

#### 4.1.2. The Mushroom Bodies

The mushroom bodies of pseudoscorpions have been described as rather reduced structures [38,39]. In general, we agree with this observation, but in contrast to Hanström [38,39], the mushroom body tracts cover the entire length of the protocerebrum in antero-posterior direction, reaching from the anterior soma cortex to the vicinities of the arcuate body (compare Figure 3E–G and Figure 428 in [39]). Anteriorly, these tracts seem to be associated with a small neuropil. In the posterior direction, the main tract splits into three branches—a lateral and two medial ones. A clear contralateral connection (mushroom body bridge) of the medial lobes as observed by Hanström [38,39] could not be identified. Corresponding to Hanström [38,39], no distinct clusters of globuli cells were identified, which are generally interpreted as a typical characteristic for mushroom bodies (e.g. [43,76]). Interestingly, no globuli cells per definition [77,78] have been found in the jumping spider *Marpissa muscosa*, although this species possesses elaborated mushroom bodies [59].

Within Arachnida, the mushroom bodies of Amblypygi (whip spiders), Thelyphonida (whip scorpions), and Araneae (spiders) are rather complex, compared to pseudoscorpions and Solifugae (sun spiders, camel spiders). In the three former taxa, globuli cells supply prominent mushroom body pedunculi and several lobes (also termed hafts), which evolved to extensive convoluted structures in Thelyphonida and Amblypygi [54,58]. Furthermore, the mushroom body is closely associated with the protocerebral glomerular neuropil, which is termed the mushroom body calyx by some authors (e.g. [55,56,58]), while others identify these structures as distinct second-order visual neuropils associated with the mushroom body [57,59,60]). In either case, the amblypygid [58] and araneaen [57,59,60] mushroom bodies receive strong input from the visual system (but note structural variation depending on species, as pointed out by Long [60]). Visual input of the solifuge mushroom body neuropil seems to be absent [79].

In addition to visual input, the mushroom bodies of amblypygids receive input from the first chemosensory integration center associated with the antenniform legs [58]. A similar observation has been made for Solifugae, where projection neurons from the chemosensory glomeruli of the malleolar system innervate the mushroom body neuropil [80].

In pseudoscorpions, we could not identify any sophisticated neuropilar structures nor protocerebral glomeruli associated with the mushroom body tracts. Additionally, the visual system is comparatively simple, lacking medial eyes. Where lateral eyes occur (as in *Chelifer cancroides*), they are suggested to serve as light detectors, but not to generate sharp images [3,81]. The most comprehensive investigation of the visual system by Lehmann and Melzer [47] does not comment on a connection between the visual neuropils and the mushroom body in pseudoscorpions.

In conclusion, the mushroom bodies of *C. cancroides* show structural similarities to those in Solifugae. Small neuropilar regions are connected via tracts to two or three lobes, lacking a mushroom body bridge (Figure 3 this study, [80]). Both taxa seem to lack visual input to the mushroom bodies [47,79], but at least solifuges receive chemosensory input via projection neurons [80]. We could show ascending tracts connecting the pedipalpal glomerular neuropil to protocerebral structures (Figure 4H). Although we could not identify the target area of these projection neurons, the mushroom bodies are good candidates to be the second-order integration center of chemosensory information. Based on these facts, the comparatively reduced mushroom bodies in *C. cancroides* as well as in solifuges might be explained by missing input from the visual system. In these taxa, the mushroom bodies might be mainly limited to chemosensory input.

### 4.2. The Glomerular Neuropil Represents a First Integration Center for Chemosensory Cues

Although the presence of chemosensory sensilla on the pedipalps of pseudoscorpions awaits its discovery, it is reasonable to interpret the glomerular neuropil within the pedipalpal neuromere as the first morphological evidence for an elaborated chemosensory pathway. We could clearly show an association of the glomerular neuropil to the sensilla on the fingers of the pedipalpal chelae and conclude this neuropil to be the first integration center of a chemosensory pathway in *C. cancroides*. A glomerular organization of chemosensory neuropils has been described for many mandibulates (e.g., insects: [82,83]; crustaceans: [83,84,85,86,87]; and myriapods: [66,88]), as well as invertebrates and even vertebrates (reviewed by, e.g. [51,52,53]). Glomerular structures are also known from other arachnid taxa, for example from the pecten neuropil of scorpions (e.g. [32,33,54,70,89]), the malleolar neuropil of Solifugae (e.g. [80,89,90]), the neuropil associated with the Haller’s organ in ticks (e.g. [91,92]), or the neuromeres of the antenniform legs of amblypygids (e.g. [54,58]) and thelyphonids (e.g. [54]). Furthermore, chemosensory pathways are mostly characterized by a chemotopic innervation pattern, where each glomerulus typically collates the axons of one particular chemoreceptor type, allowing for spatial segregation of chemical cues (e.g. [75,93,94]). Although we cannot demonstrate a definite chemotopy, the homogenous innervation of all glomeruli in the differential labeling of the fingers (Figure 6 and Figure 7) hints toward such a projection pattern of chemosensory afferents.

Further elements of a typical mandibulate chemosensory system associated with the first pair of antennae are chemosensory projection neurons (see above, section “The Mushroom Bodies”) and chemosensory local interneurons [75,83,95]. The latter link the chemosensory glomeruli and synapse with the receptor neurons and projection neurons. The chemosensory glomeruli in *C. cancroides* are innervated by axons from the ventral cortex of the pedipalpal neuromere, which we interpret as chemosensory local interneurons (Figure 4I).

### 4.3. The Layered Neuropil Represents a First Integration Center for Mechanosensory Cues

In Mandibulata, the mechanosensory neuropils associated with the first antennae are characterized by a deutocerebral bilaterally paired neuropil, and they are clearly separated from deutocerebral chemosensory centers. These deutocerebral mechanosensory neuropils often show a striate or palisade shape and contralateral connections (e.g. [75,88,96]). In Myriapoda, this neuropil has been termed *masse lamelleuse* [97] and accordingly *corpus lamellosum* [88,98].

Additionally, mechanosensory integration centers typically show a somatotopic innervation pattern in Mandibulata (e.g., Myriapoda: [88,99]; Crustacea: [100,101]; and Hexapoda: [102,103,104]). The position of the sensilla on the body surface is reflected by its target region in the mechanosensory neuropil. Similarly, a topographic projection pattern of mechanosensory afferents has been demonstrated in the spider *Cupiennius salei* [105,106,107] and the scorpion *Heterometrus fulvipes* [108]. Based on these structural similarities (layered arrangement, topographic innervation pattern), we interpret the layered neuropil in the pedipalpal neuromere of *C. cancroides* as a mechanosensory integration center.

## 5. Conclusions

The data presented in this contribution give the first morphological evidence for a sensory system related to the pedipalps of pseudoscorpions, which resembles the general appearance of the mechano- as well as chemosensory pathway associated with the first antennae in Mandibulata. Due to its elaborated equipment with sensory sensilla and the associated sensory neurons, the pedipalps can clearly be classified as the most important sensory organ in pseudoscorpions.

Chemosensory systems within Arachnida show very similar arrangements of neuropils, but have evolved in different body regions, which precludes homology of these structures. In fact, the similarities in the arrangement of chemosensory pathways of different evolutionary origin, for example, the formation of glomerular structures, have been interpreted as functional prerequisites for this sensory modality (e.g. [51,52,53,83]).

However, it is still unclear which chemical cues are relevant. Although several (mostly anecdotal) observations support involvement of chemosensory cues in pseudoscorpion ecology, scientific evidence is mostly missing [109]. Along these lines, it remains unclear to which extent pseudoscorpions detect airborne signals or depend on contact-chemoreception. Electrophysiological approaches combined with behavioral assays as well as a description of the morphology of the chemosensory sensilla on the pedipalps will help to answer these questions.

## Data Availability

The data presented in this study are available in the article.

## References

[1] Harvey M.S. (2007). The smaller arachnid orders: Diversity, descriptions and distributions from Linnaeus to the present (1758 to 2007). Zootaxa.

[2] Levi H.W. (1953). Observation on two species of pseudoscorpions. Can. Entomol..

[3] Weygoldt P. (1969). Biology of Pseudoscorpions.

[4] Brach V. (1978). Social behavior in the pseudoscorpion *Paratemnus elongatus* (Banks) (Pseudoscorpionida, Atemnidae). Insect Soc..

[5] Zeh D.W. (1987). Life history consequences of sexual dimorphism in a chernetid pseudoscorpion. Ecology.

[6] Zeh J.A., Zeh D.W. (1990). Cooperative Foraging for Large Prey by *Paratemnus Elongatus* (Pseudoscorpionida, Atemnidae). J. Arachnol..

[7] Weygoldt P. (1966). Vergleichende Untersuchungen zur Fortpflanzungsbiologie der Pseudoscorpione. Beobachtungen über das Verhalten, die Samenübertragungsweisen und die Spermatophoren einiger einheimischer Arten. Zeitschrift Morphologie Ökologie Tiere.

[8] Beier M. (1948). Phoresie und Phagophilie bei Pseudoscorpionen. Oesterreich. Zool. Z..

[9] Poinar G.O., Curcic B.P.M., Cokendolpher J.C. (1998). Arthropod Phoresy Involving Pseudoscorpions in the Past and Present. Acta Arachnol..

[10] Zeh D.W., Zeh J.A. (1992). On the function of Harlequin Beetle-riding in the pseudoscorpion, *Cordylochernes scorpioides* (Pseudoscorpionida: Chernetidae). J. Arachnol..

[11] Zeh D.W., Zeh J.A. (1992). Failed Predation or Transportation? Causes and Consequences of Phoretic Behavior in the Pseudoscorpion *Dinocheirus arizonensis* (Pseudoscorpionida: Chernetidae). J. Insect Behav..

[12] Zeh J.A., Newcomer S.D., Zeh D.W. (1998). Polyandrous females discriminate against previous mates. Proc. Natl. Acad. Sci. USA.

[13] Bonilla M.M., Zeh D.W., White A.M., Zeh J.A. (2011). Discriminating Males and Unpredictable Females: Males Bias Sperm Allocation in Favor of Virgin Females. Ethology.

[14] Proctor H.C. (1993). Mating biology resolves trichotomy for cheliferoid pseudoscorpions (Pseudoscorpionida, Cheliferoidea). J. Arachnol..

[15] Vachon M. (1938). Recherches anatomiques et biologiques sur la reproduction et le développement des Pseudoscorpions. Ann. Sci. Nat. Zool. Biol. Anim..

[16] Harvey M.S. (2014). A review and redescription of the cosmopolitan pseudoscorpion *Chelifer cancroides* (Pseudoscorpiones: Cheliferidae). J. Arachnol..

[17] Weygoldt P. (1970). Vergleichende Untersuchungen zur Fortpflanzungsbiologie der Pseudoscorpione II. J. Zool. Syst. Evol. Res..

[18] Legg G. (1973). Spermatophore formation in the pseudoscorpion *Chthonius ischnocheles* (Chthoniidae). J. Zool..

[19] Legg G. (2009). Taxonomy and the Dangers of Sex with special Reference to Pseudoscorpions. Adv. Arachnol. Dev. Biol..

[20] Judson M.L.I. (1992). *Roncocreagris murphyorum* n. sp. and *Occitanobisium nanum* (Beier) n. comb. (Neobisiidae) from Iberia, with notes on the sternal glands of pseudoscorpions (Chelonethi). Bull. Br. Arachnol. Soc..

[21] Judson M.L.I. (2007). A new and endangered pseudoscorpion of the genus *Lagynochthonius* (Arachnida, Chelonethi, Chthoniidae) from a cave in Vietnam, with notes on chelal morphology and the composition of the Tyrannochthoniini. Zootaxa.

[22] Judson M.L.I. (2016). Pseudoscorpions (Arachnida, Chelonethi) in Mexican amber, with a list of extant species associated with mangrove and Hymenaea trees in Chiapas. Bol. Soc. Geol. Mex..

[23] Judson M.L.I. (2017). A new subfamily of Feaellidae (Arachnida, Chelonethi, Feaelloidea) from Southeast Asia. Zootaxa.

[24] Reboleira A.S.P.S., Zaragoza J.A., Goncalves F., Oromí P. (2010). *Titanobochica*, surprising discovery of a new cave-dwelling genus from southern Portugal (Arachnida: Pseudoscorpiones: Bochicidae). Zootaxa.

[25] Mahnert V. (2011). A nature’s treasury: Pseudoscorpion diversity of the Canary Islands, with the description of nine new species (Pseudoscorpiones, Chthoniidae, Cheiridiidae) and new records. Revista Ibérica de Aracnologia.

[26] Mahnert V. (2014). Pseudoscorpions (Arachnida: Pseudoscorpiones) from the Galapagos Islands (Ecuador). Rev. Suisse Zool..

[27] Moro S.D., Geethabali (1985). Distribution of cuticular sensory hairs on the legs and whip of *Thelyphonus indicus stoliczka* (Arachnida Uropygi). Ital. J. Zool..

[28] Igelmund P. (1987). Morphology, sense organs, and regeneration of the forelegs (whips) of the whip spider *Heterophrynus elaphus* (Arachnida, Amblypygi). J. Morphol..

[29] Schultz J.W. (1989). Morphology of locomotor appendages in Arachnida: Evolutionary trends and phylogenetic implications. Zool. J. Linnean Soc..

[30] Weygoldt P. (2000). Whip Spiders. Their Biology, Morphology and Systematics (Chelicerata: Amblypygi).

[31] Carthy J.D. (1966). Fine structure and function of the sensory pegs on the scorpion pecten. Experientia.

[32] Wolf H. (2008). The pectine organs of the scorpion, *Vaejovis spinigerus*: Structure and (glomerular) central projections. Arthropod Struct. Dev..

[33] Wolf H. (2017). Scorpions pectines—Idiosyncratic chemo- and mechanosensory organs. Arthropod Struct. Dev..

[34] Brownell P.H., Farley R.D. (1974). The organization of the malleolar sensory system in the solpugid, *Chanbria* sp. Tissue Cell.

[35] Lehmann T., Melzer R.R., Hörnig M.K., Michalik P., Sombke A., Harzsch S., Schmidt-Rhaesa A., Harzsch S., Purschke G. (2016). Arachnida (excluding Scorpiones). Structure and Evolution of Invertebrate Nervous Systems.

[36] Hilton W.A. (1913). The nervous system of Chelifer. J. Entomol. Zool..

[37] Hilton W.A. (1931). Nervous system and sense organs. Pseudoscorpionida. J. Entomol. Zool..

[38] Hanström B. (1919). Zur Kenntnis des Zentralen Nervensystems der Arachnoiden und Pantopoden Nebst Schlussfolgerungen Betreffs der Phylogenie der Genannten Gruppen. Ph.D. Thesis.

[39] Hanström B. (1928). Vergleichende Anatomie des Nervensystems der Wirbellosen Tiere: Unter Berücksichtigung seiner Funktion.

[40] Strausfeld N.J. (2012). Arthropod Brains; Evolution, Functional Elegance, and Historical Significance.

[41] Wolff G.H., Strausfeld N.J., Schmidt-Rhaesa A., Harzsch S., Purschke G. (2016). The Insect Brain: A commentated primer. Structure and Evolution of Invertebrate Nervous Systems.

[42] Heisenberg M. (2003). Mushroom body memoir: From maps to models. Nat. Rev. Neurosci..

[43] Strausfeld N.J., Sinakevitch I., Brown S.M., Farris S.M. (2009). Ground plan of the insect mushroom body: Functional and evolutionary implications. J. Comp. Neurol..

[44] Campbell R.A.A., Turner G.C. (2010). The mushroom body. Curr. Biol..

[45] Aso Y., Hattori D., Yu Y., Johnston R.M., Iyer N.A., Ngo T.T.B., Dionne H., Abbott L.F., Axel R., Tanimoto H. (2014). The neuronal architecture of the mushroom body provides a logic for associative learning. eLife.

[46] Stopfer M. (2014). Central processing in the mushroom bodies. Curr. Opin. Insect Sci..

[47] Lehmann T., Melzer R.R. (2018). A tiny visual systemdretinula axons and visual neuropils of *Neobisium carcinoides* (Hermann, 1804)(Chelicerata, Arachnida, Pseudoscorpiones). Zoologischer Anzeiger.

[48] Boissin L., Cazal M. (1969). Étude du système nerveux et des glandes endocrines céphaliques de l’adulte femelle *d’Hysterochelifer meridianus* (L. Koch) (Arachnide, Pseudoscorpion, Cheliferidae). Bull. Soc. Zool. France.

[49] Maurer M., Hladik J., Iliffe T.M., Stemme T. (2019). Histaminergic interneurons in the ventral nerve cord: Assessment of their value for Euarthropod phylogeny. Zool. Lett..

[50] Weygoldt P. (1964). Vergleichend-embryologische Untersuchungen an Pseudoscorpionen (Chelonethi). Z. Morphol. Oekol. Tiere.

[51] Strausfeld N.J., Hildebrand J.G. (1999). Olfactory systems: Common design, uncommon origins?. Curr. Opin. Neurol..

[52] Eisthen H.L. (2002). Why Are Olfactory Systems of Different Animals So Similar?. Brain Behav. Evol..

[53] Ache B.W., Young J.M. (2005). Olfaction: Diverse Species, Conserved Principles. Neuron.

[54] Babu K.S. (1965). Anatomy of the central nervous system of arachnids. Zool. Jb. Anat..

[55] Hill D.E. (1975). The Structure of the Central Nervous System of Jumping Spiders of the Genus *Phidippus* (Araneae: Salticidae). Master’s Thesis.

[56] Babu K.S., Barth F.G. (1984). Neuroanatomy of the central nervous system of the wandering spider, *Cupiennius salei* (Arachnida, Araneida). Zoomorphology.

[57] Strausfeld N.J., Barth F.G. (1993). Two visual systems in one brain: Neuropils serving the secondary eyes of the spider *Cupiennius salei*. J. Comp. Neurol..

[58] Sinakevitch I., Long S.M., Gronenberg W. (2021). The central nervous system of whip spiders (Amblypygi): Large mushroom bodies receive olfactory and visual input. J. Comp. Neurol..

[59] Steinhoff P.O.M., Uhl G., Harzsch S., Sombke A. (2020). Visual pathways in the brain of the jumping spider *Marpissa muscosa*. J. Comp. Neurol..

[60] Long S.M. (2021). Variations on a theme: Morphological variation in the secondary eye visual pathway across the order of Araneae. J. Comp. Neurol..

[61] Schneeberg K., Bauernfeind R., Pohl H. (2017). Comparison of cleaning methods for delicate insect specimens for scanning electron microscopy. Microsc. Res. Tech..

[62] Michels J., Büntzow M. (2010). Assessment of Congo red as a fluorescence marker for the exoskeleton of small crustaceans and the cuticle of polychaetes. J. Microsc..

[63] Stemme T., Eickhoff R., Bicker G. (2014). Olfactory projection neuron pathways in two species of marine Isopoda (Peracarida, Malacostraca, Crustacea). Tissue Cell.

[64] Harzsch S., Hansson B.S. (2008). Brain architecture in the terrestrial hermit crab *Coenobita clypeatus* (Anomura, Coenobitidae), a crustacean with a good aerial sense of smell. BMC Neurosci..

[65] Groh C., Rössler W. (2011). Comparison of microglomerular structures in the mushroom body calyx of neopteran insects. Arthropod Struct. Dev..

[66] Sombke A., Harzsch S., Hansson B.S. (2011). Organization of deutocerebral neuropils and olfactory behavior in the centipede *Scutigera coleoptrata* (Linnaeus, 1758) (Myriapoda: Chilopoda). Chem. Senses.

[67] Stemme T., Iliffe T.M., von Reumont B.M., Koenemann S., Harzsch S., Bicker G. (2013). Serotonin-immunoreactive neurons in the ventral nerve cord of Remipedia (Crustacea): Support for a sister group relationship of Remipedia and Hexapoda?. BMC Evol. Biol..

[68] Fabian-Fine R., Volknandt W., Seyfarth E.A. (1999). Peripheral synapses at identifiable mechanosensory neurons in the spider *Cupiennius salei*: Synapsin-like immunoreactivity. Cell Tissue Res..

[69] Steinhoff P.O.M., Sombke A., Liedtke J., Schneider J.M., Harzsch S., Uhl G. (2017). The synganglion of the jumping spider *Marpissa muscosa* (Arachnida: Salticidae): Insights from histology, immunohistochemistry and microCT analysis. Arthropod Struct. Dev..

[70] Drozd D., Wolf H., Stemme T. (2020). Structure of the pecten neuropil pathway and its innervation by bimodal peg afferents in two scorpion species. PLoS ONE.

[71] Strausfeld N.J., Weltzien P., Barth F.G. (1993). Two visual systems in one brain: Neuropils serving the principal eyes of the spider *Cupiennius salei*. J. Comp. Neurol..

[72] Loesel R., Nässel D.R., Strausfeld N.J. (2002). Common design in a unique midline neuropil in the brains of arthropods. Arthropod Struct. Dev..

[73] Loesel R., Seyfarth E.A., Bräunig P., Agricola H.J. (2011). Neuroarchitecture of the arcuate body in the brain of the spider *Cupiennius salei* (Araneae, Chelicerata) revealed by allatostatin-, proctolin-, and CCAP-immunocytochemistry and its evolutionary implications. Arthropod Struct. Dev..

[74] Wolf H., Schmidt-Rhaesa A., Harzsch S., Purschke G. (2019). Scorpiones. Structure and Evolution of Invertebrate Nervous Systems.

[75] Loesel R., Wolf H., Kenning M., Harzsch S., Sombke A., Minelli A., Boxshall G., Fusco G. (2013). Architectural principles and evolution of the arthropod central nervous system. Arthropod Biology and Evolution.

[76] Wolff G.H., Strausfeld N.J. (2016). Genealogical correspondence of a forebrain centre implies an executive brain in the protostome-deuterostome bilaterian ancestor. Philos. Trans. R. Soc..

[77] Kenyon F.C. (1896). The meaning and structure of the so-called “mushroom bodies” of the hexapod brain. Am. Nat..

[78] Richter S., Loesel R., Purschke G., Schmidt-Rhaesa A., Scholtz G., Stach T., Vogt L., Wanninger A., Brenneis G., Döring C. (2010). Invertebrate neurophylogeny: Suggested terms and definitions for a neuroanatomical glossary. Front. Zool..

[79] Lehmann T., Melzer R.R. (2021). Outsourcing a visual neuropil—The central visual system of the median eyes of *Galeodes granti* Pocock, 1903 (Arachnida: Solifugae). Arthropod Struct. Dev..

[80] Sombke A., Klann A.E., Lipke E., Wolf H. (2019). Primary processing neuropils associated with the malleoli of camel spiders (Arachnida, Solifugae): A re-evaluation of axonal pathways. Zool. Lett..

[81] Demoll R. (1917). Die Sinnesorgane der Arthropoden ihr Bau und ihre Funktion.

[82] Hansson B.S., Anton S. (2000). Function and Morphology of the Antennal Lobe: New Developments. Annu. Rev. Entomol..

[83] Schachtner J., Schmidt M., Homberg U. (2005). Organization and evolutionary trends of primary olfactory brain centers in Tetraconata (Crustacea + Hexapoda). Arthropod Struct. Dev..

[84] Schmidt M., Mellon D., Breithaupt T., Thiel M. (2011). Neuronal processing of chemical information in crustaceans. Chemical Communication in Crustaceans.

[85] Stegner M.E.J., Richter S. (2011). Morphology of the brain in *Hutchinsoniella macracantha* (Cephalocarida, Crustacea). Arthropod Struct. Dev..

[86] Stemme T., Iliffe T.M., Bicker G., Harzsch S., Koenemann S. (2012). Serotonin immunoreactive interneurons in the brain of the Remipedia: New insights into the phylogenetic affinities of an enigmatic crustacean taxon. BMC Evol. Biol..

[87] Harzsch S., Krieger J. (2018). Crustacean olfactory systems: A comparative review and a crustacean perspective on olfaction in insects. Prog. Neurobiol..

[88] Sombke A., Lipke E., Kenning M., Müller C.H.G., Hansson B.S. (2012). Comparative analysis of deutocerebral neuropils in Chilopoda (Myriapoda): Implications for the evolution of the arthropod olfactory system and support for the Mandibulata concept. BMC Neurosci..

[89] Brownell P.H. (1998). Glomerular Cytoarchitectures in chemosensory Systems of Arachnids. Ann. N. Y. Acad. Sci..

[90] Strausfeld N.J., Reisenman C.E. (2009). Dimorphic Olfactory Lobes in the Arthropoda. Ann. N. Y. Acad. Sci..

[91] Hummel N.A., Li A.Y., Witt C.M. (2007). Serotonin-like immunoreactivity in the central nervous system of two ixodid tick species. Exp. Appl. Acarol..

[92] Menezes K.M.F., de Oliveira Filho J.G., Ferreira L.L., Borges L.M.F. (2021). First neuronal projection from Haller’s organ to the synganglion and three-dimensional reconstruction of *Amblyomma sculptum* olfactory lobe. Tick Tick Borne Dis..

[93] Hansson B.S., Christensen T.A., Hansson B.S. (1999). Functional characteristics of the antennal lobe. Insect Olfaction.

[94] Ignell R., Anton S., Hansson B.S. (2001). The antennal lobe of Orthoptera–Anatomy and Evolution. Brain Behav. Evol..

[95] Polanska M.A., Tuchina O., Agricola H., Hansson B.S., Harzsch S. (2012). Neuropeptide complexity in the crustacean central olfactory pathway: Immunolocalization of A-type allatostatins and RFamide-like peptides in the brain of a terrestrial hermit crab. Mol. Brain.

[96] Strausfeld N.J. (1998). Crustacean–insect relationships: The use of brain characters to derive phylogeny amongst segmented invertebrates. Brain Behav. Evol..

[97] Saint-Rémy G. (1887). Contribution a l’étude du cerveau chez les arthropods trachéates. Arch. Zool. Exp. Gen..

[98] Fahlander K. (1938). Beiträge zur Anatomie und systematischen Einteilung der Chilopoden. Zoologiska Bidrag från Uppsala.

[99] Nguyen Duy-Jacquemin M.N., Arnold G. (1991). Spatial organization of the antennal lobe in *Cylindroiulus punctatus* (Leach) (Myriapoda: Diplopoda). Int. J. Insect Morphol. Embryo..

[100] Schmidt M., Van Ekeris L., Ache B.W. (1992). Antennular projections to the midbrain of the spiny lobster. I. Sensory innervation of the lateral and medial antennular neuropils. J. Comp. Neurol..

[101] Tuchina O., Koczan S., Harzsch S., Rybak J., Wolff G., Strausfeld N.J., Hansson B.S. (2015). Central projections of antennular chemosensory and mechanosensory afferents in the brain of the terrestrial hermit crab (*Coenobita clypeatus*; Coenobitidae, Anomura). Front. Neuroanat..

[102] Pflüger H.J., Bräunig P., Hustert R. (1988). The organization of mechanosensory neuropiles in locust thoracic ganglia. Phil. Trans. R. Soc. Lond. B..

[103] Newland P.L. (1991). Morphology and somatotopic organisation of the central projections of afferents from tactile hairs on the hind leg of the locust. J. Comp. Neurol..

[104] Newland P.L., Rogers S.M., Gaaboub I., Matheson T. (2000). Parallel somatotopic maps of gustatory and mechanosensory neurons in the central nervous system of an insect. J. Comp. Neurol..

[105] Babu K.S., Barth F.G. (1989). Central nervous projections of mechanoreceptors in the spider *Cupiennius salei* Keys. Cell Tissue Res..

[106] Anton S., Barth F.G. (1993). Central nervous projection patterns of trichobothria and other cuticular sensilla in the wandering spider *Cupiennius salei* (Arachnida, Araneae). Zoomorphology.

[107] Gorb S.N., Anton S., Barth F.G. (1993). Central projections of cheliceral mechanoreceptors in the spider *Cupiennius salei* (Arachnida, Araneae). J. Morphol..

[108] Babu K.S., Sreenivasulu K., Sekhar V. (1993). Sensory projections of identified coxal hair sensilla of the scorpion *Heterometrus fulvipes* (Scorpionidae). J. Biosci..

[109] Kirchmair G., Raspotnig G. (2021). Mating behavior of *Dactylochelifer latreillii latreillii* (Pseudoscorpiones: Cheliferidae): A quantitative study. J. Arachnol..

